# A Synergistic Effect between Plasma Dickkopf-1 and Obstructive Coronary Artery Disease on the Prediction of Major Adverse Cardiac Events in Patients with Angina: An Observational Study

**DOI:** 10.3390/biom12101408

**Published:** 2022-10-02

**Authors:** Yu-Hsuan Li, Min-Huan Wu, Wen-Jane Lee, I-Te Lee

**Affiliations:** 1 Division of Endocrinology and Metabolism, Department of Internal Medicine, Taichung Veterans General Hospital, Taichung 407219, Taiwan; 2 Department of Computer Science & Information Engineering, National Taiwan University, Taipei 106216, Taiwan; 3 Bachelor of Science in Senior Wellness and Sport Science, Tunghai University, Taichung 407224, Taiwan; 4 Senior Life and Innovation Technology Center, Tunghai University, Taichung 407224, Taiwan; 5 Life Science Research Center, Tunghai University, Taichung 407224, Taiwan; 6 Department of Medical Research, Taichung Veterans General Hospital, Taichung 407219, Taiwan; 7 School of Medicine, National Yang Ming Chiao Tung University, Taipei 112304, Taiwan; 8 School of Medicine, Chung Shan Medical University, Taichung 402306, Taiwan

**Keywords:** coronary artery disease, death, dickkopf-1, major adverse cardiac events, myocardial infarction, obstruction, stroke

## Abstract

The canonical β-catenin-dependent wingless (Wnt) pathway is associated with endothelial function. We examined the effect of plasma dickkopf-1 (DKK-1), an inhibitor of the Wnt pathway, on the prediction of major adverse cardiac events (MACEs). We enrolled patients who had undergone selective coronary angiography for angina. DKK-1 levels were determined using plasma collected at the outpatient visit after fasting. MACEs served as the primary endpoint. All 470 enrolled patients were divided into four groups according to their median plasma DKK-1 levels and the presence of obstructive coronary artery disease (CAD). Forty-eight patients reached the primary endpoint during a median follow-up time of 4.8 years. Kaplan–Meier survival analysis indicated that the group with high DKK-1 and obstructive CAD had a significantly higher mortality rate than the other three groups (log-rank test *p* = 0.001). Compared with the low plasma DKK-1 without significant coronary obstruction group, the high DKK-1 with obstructive CAD group had a hazard ratio of 10.640 (95% confidence interval: 1.350–83.874) for MACEs, as determined by multivariable Cox proportional hazard regression analysis. In conclusion, we observed a synergistic effect between high plasma DKK-1 and obstructive CAD on the prediction of MACEs in patients with angina.

## 1. Introduction

The global number of patients with ischemic heart disease (IHD) is increasing, and IHD remains a major health burden worldwide [1,2]. According to a report from the Global Burden of Diseases, Injuries, and Risk Factors Study in 2017, the leading cause of death was IHD [3], and in 2019, the largest burden of disability was caused by IHD in people aged ≥ 50 years old [4]. However, traditional risk factors cannot predict the exact risk of cardiovascular events and mortality, and the identification of new biomarkers is warranted to improve upon this limitation [5]. 

Dickkopf-1 (DKK-1) is a secreted glycoprotein that acts as an inhibitor of the canonical β-catenin-dependent wingless (Wnt) pathway [6]. Wnt proteins regulate several physiological processes, including cell proliferation, differentiation, migration, and apoptosis [7]. Since the Wnt pathway is involved in cardiovascular development and endothelial function [8,9], increased expression of DKK-1 might be associated with the development of atherosclerosis [10]. 

Admission serum levels of DKK-1 in patients with acute coronary syndrome have been reported to be associated with major adverse cardiac events (MACEs), a composite of cardiovascular death, myocardial infarction (MI), or stroke [11]. DKK-1 is involved in the platelet-mediated endothelial cell activation induced by inflammation [12]. Notably, the coagulating process can induce the secretion of DKK-1, and serum DKK-1 levels have been reported to be higher in patients with angina than in controls. However, there was no significant difference in plasma DKK-1 levels between patients with angina and controls [12]. The association between plasma DKK-1 and cardiovascular outcomes remains unexplored. Therefore, we hypothesized that the DKK-1 concentration in plasma could predict MACEs.

## 2. Materials and Methods

### 2.1. Study Participants

This observational cohort study was conducted at Taichung Veterans General Hospital between January 2010 and March 2018. The study complied with the guidelines of the Declaration of Helsinki and was approved by the Institutional Review Board of Taichung Veterans General Hospital. Written informed consent was obtained from every participant before the start of any procedure. The inclusion criteria for patient enrollment were (a) a history of angina, (b) a diagnosis of IHD based on at least a noninvasive examination, and (c) a selective coronary angiography performed in our hospital. The exclusion criteria were as follows: (a) age < 20 years, (b) a history of known diabetes mellitus (DM), (c) a history of known cancer, (d) current medication for any autoimmune disease or any psychiatric disease, (e) current acute infection, (f) symptomatic congestive heart failure ≥ Class III based on the criteria of the New York Heart Association [13], and (g) pregnancy.

### 2.2. Procedures

After patients were discharged in a stable condition, the eligible patients were scheduled for an outpatient follow-up appointment. We enrolled 470 patients between January 2010 and December 2016. Following anthropometric measurements in the outpatient interview, fasting blood samples were collected. We recorded the coronary angiography and medications by reviewing electronic medical records. After the baseline assessment, all of the participants were followed up through March 2018. Death due to any cause or the first episode of nonfatal MI or nonfatal stroke was recorded as the primary endpoint of MACEs based on electronic medical records from our hospital. For patients without any endpoint recorded before February 2018, we arranged a telephone call interview in March 2018, and information on the primary endpoint was collected from the patients themselves or from their immediate family members. Among all of the enrolled participants, 431 (91.7%) completed follow-up, and the data accuracy was confirmed using death registration information obtained from the Ministry of Health and Welfare, Executive Yuan, Taiwan. In the 39 (8.3%) participants who were lost to follow-up, we confirmed death using death registration information. There was no significant difference in the mortality rate between the participants who were followed up and those who were lost to follow-up (*p* = 0.351).

### 2.3. Laboratory Assessments

The blood samples, which were drawn from the antecubital vein, were collected in tubes containing EDTA. After the samples were centrifugated at 3000 RPM for 10 minutes at 4 °C, the plasma was separated and stored at −80 °C. Plasma samples were prepared to detect glucose, insulin, N-terminal pro-brain natriuretic peptide (NT-proBNP), and DKK-1 levels. Human DKK-1 was measured using a commercial immunoassay kit (R&D Systems, Minneapolis, MN, USA). The precision of the DKK-1 measurements was indicated by an intra-assay coefficient of variation (CV) of 2.6% and an inter-assay CV of 8.1%. The minimum detectable level of the DKK-1 measurement was 0.419 pg/mL. Platelet counts were determined using an automated cell counter (Sysmex, Kobe, Hyogo, Japan). Human NT-proBNP was measured using a commercial immunoassay kit (Cusabio Biotech Co, Wuhan, China). Serum samples were prepared to measure creatinine levels, C-reactive protein (CRP), and the lipid profile. CRP was determined using an ELISA kit (R&D Systems, Minneapolis, MN, USA). A morning urine sample was collected for the measurement of albumin and creatinine to calculate the urine albumin to creatinine ratio (UACR).

The homeostasis model assessment of insulin resistance (HOMA-IR) index was calculated using the following formula: fasting insulin (µIU/mL) × fasting glucose (mmol/L)/22.5 for the quantitative evaluation of insulin resistance [14]. According to the Modification of Diet in Renal Diseases equation, the estimated glomerular filtration rate (eGFR) was calculated using the following formula: 186 × (serum creatinine [mg/dL])^−1.154^ × (age [years])^−0.203^ (× 0.742, if female) [15]. Chronic kidney disease (CKD) was defined as an eGFR < 60 mL/min/1.73 m^2^ [15]. UACR was calculated as the ratio of urine albumin (mg) to urine creatinine (g), and albuminuria was defined as UACR ≥ 300 mg/g [15]. Hypertension was defined at baseline when any of the following conditions were present: (a) a history of antihypertensive agent use, (b) systolic blood pressure ≥ 140 mmHg, or (c) diastolic blood pressure ≥ 90 mmHg on the day of the interview. The Framingham Risk Score (FRS) was calculated as a standard risk factor for MACEs [16]. CAD was defined as an obstructive lesion of the coronary artery at baseline when one or more of the following conditions were present: (a) a history of MI, (b) a history of coronary revascularization, or (c) a coronary lesion with lumen narrowing ≥ 50% according to angiography.

### 2.4. Statistical Analysis

Continuous variables are presented as the mean ± standard deviation, and we used the Kolmogorov–Smirnov test to examine the normality of the data. We used the t-test or the Mann–Whitney U test between two groups and analysis of variance (ANOVA) or the Kruskal–Wallis test among more than two groups to ascertain the significance of the difference in the continuous variables. Categorical variables are presented as numbers (with percentages). We used chi-squared tests to ascertain the significance of the difference in the categorical variables. 

The risk of MACEs was examined by Kaplan–Meier survival analysis among the four groups, and statistically significant differences were tested by the log-rank test. The improvement in primary-endpoint prediction by adding the variable of DKK-1 was assessed by examining the increases in Harrell’s concordance index (C index) [17]. We used the integrated discrimination improvement (IDI) and continuous net reclassification improvement (NRI) to ascertain the significance of the difference in the performance of the model adding DKK-1 [18].

Multivariable Cox proportional hazards regressions were performed to evaluate the risk of MACEs according to the groups categorized by plasma DKK-1 level and CAD status. A two-sided *p* value < 0.05 was considered to be statistically significant. Statistical analysis was conducted using SPSS v22.0 (IBM, Armonk, NY, USA) and R software v3.4.

## 3. Results

**Figure 1:**
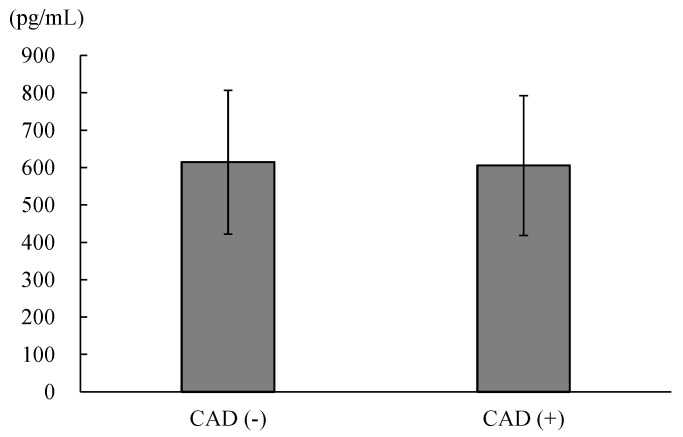
The comparison of the plasma concentrations of dickkopf-1 between patients without coronary artery disease (CAD) and those with CAD (614 ± 192 vs. 606 ± 187, *p* = 0.622).

**Figure 2:**
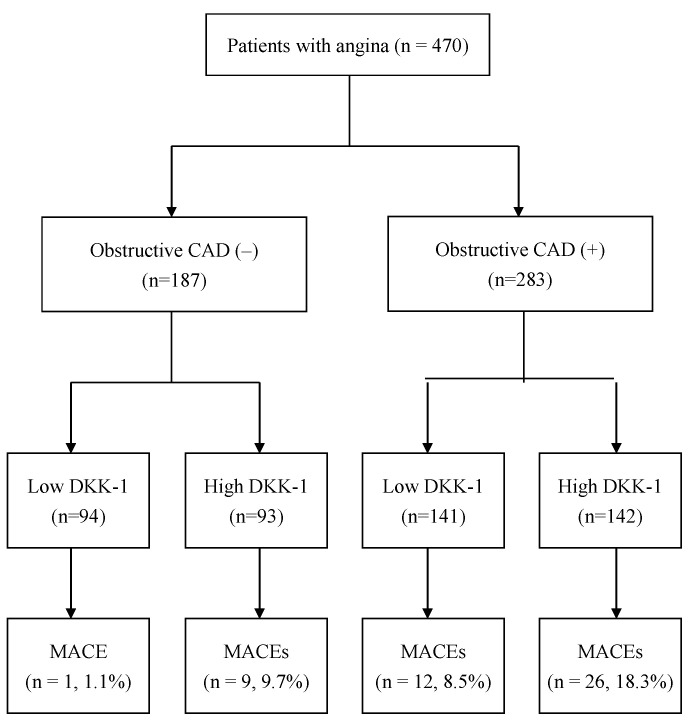
Flow diagram of the enrollment of the study participants. Abbreviation: CAD = coronary artery disease, DKK-1 = dickkopf-1, and MACEs = major adverse cardiac events.

Among the 470 patients enrolled in the present study, there were 187 patients without CAD and 283 patients with CAD. There was no significant difference in the plasma DKK-1 levels between patients without CAD and those with CAD (614 ± 192 vs. 606 ± 187 pg/mL, *p* = 0.622; Figure 1). We further divided all of the enrolled patients into four groups categorized according to their plasma DKK-1 level and CAD status. The cutoff point between low DKK-1 and high DKK-1 was the median value of 610 pg/mL (Figure 2). There was no significant difference in the duration between coronary angiography and outpatient interview: 13 days (interquartile range (IQR), 9–17 days) in the low DKK-1 without CAD group, 14 days (IQR, 10–20 days) in the high DKK-1 without CAD group, 11 days (IQR, 9–16 days) in the low DKK-1 with CAD group, and 13 days (IQR, 9–24 days) in the high DKK-1 with CAD group. There was no significant difference in the duration between coronary angiography and outpatient visits among groups (*p* = 0.146). 

The baseline characteristics of these four groups are shown in Table 1. There were significant positive trends in age (*p* < 0.001 among the four groups), the proportion of current smokers (*p* = 0.044 among the four groups), and serum CRP (*p* = 0.003 among the four groups) from the low DKK-1 without CAD group to the high DKK-1 with CAD group. There were higher proportions of males (*p* < 0.001 among the four groups), hypertension (*p* = 0.033 among the four groups), and the use of statins (*p* < 0.001 among the four groups) in the patients with CAD than those without CAD. The eGFR (*p* < 0.001) and lipid profile, including total cholesterol (*p* < 0.001), high-density lipoprotein (HDL) cholesterol (*p* < 0.001), and triglycerides (*p* = 0.042), were significantly different among these four groups.

**Figure 3:**
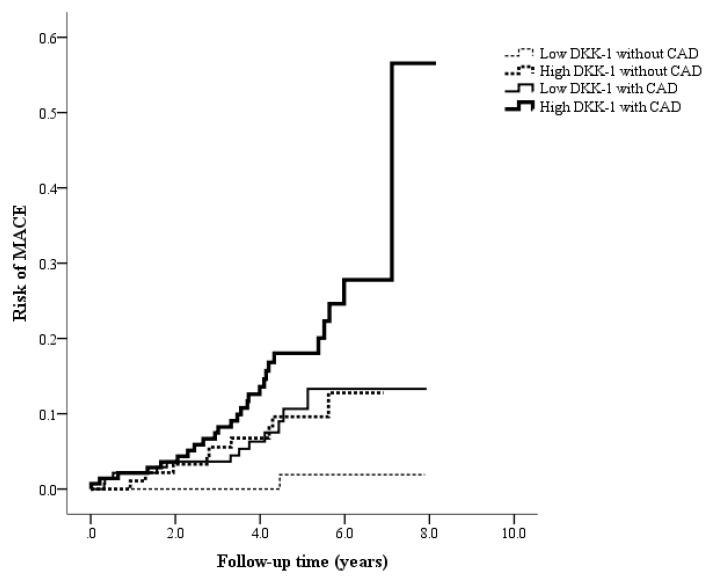
Kaplan–Meier curves showing the risk of major adverse cardiac events (MACEs) across the four groups, categorized using the median dickkopf-1 (DKK-1) value of 610 pg/mL and coronary artery disease (CAD) status (log-rank test: *p* = 0.001).

During the median follow-up time of 4.8 years, a total of 48 MACEs occurred. All the patients were divided into four groups (Figure 2): 94 patients were in the low DKK-1 without CAD group (1 MACE, 1.1%), 93 patients were in the high DKK-1 without CAD group (9 MACEs, 9.7%), 141 patients were in the low DKK-1 with CAD group (12 MACEs, 8.5%), and 142 patients were in the high DKK-1 with CAD group (26 MACEs, 18.3%, Figure 2). Figure 3 shows that the incidence of MACEs was significantly different among the four groups: MACE risk was the lowest in the low DKK-1 without CAD group and the highest in the high DKK-1 with CAD group according to Kaplan–Meier survival analysis (log-rank test: *p* = 0.001).

**Figure 4:**
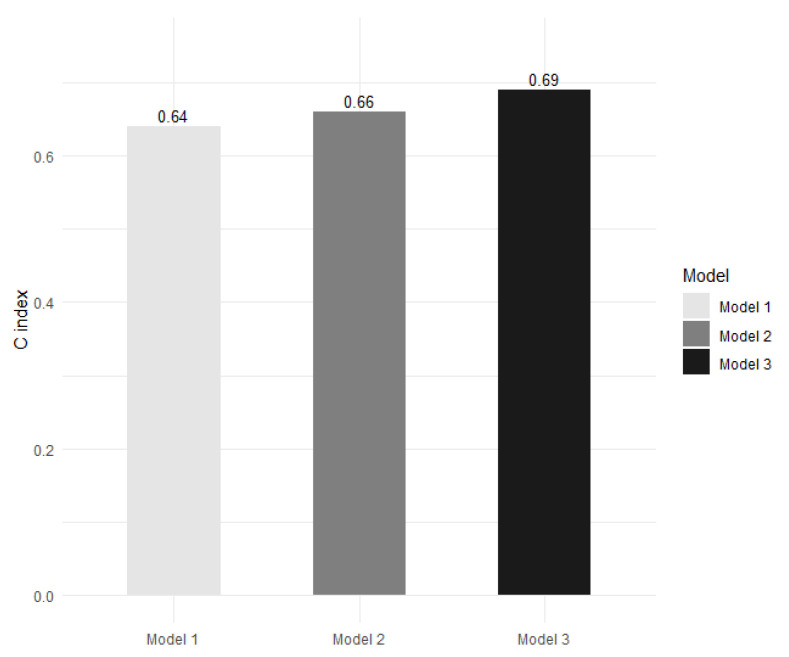
The Harrell’s concordance index (C index) for the prediction of major adverse cardiac events in the Framingham Risk Score (FRS)-alone model (model 1), in the FRS and coronary artery disease (CAD) model (model 2), and in the model adding dickkopf-1 (DKK-1) to FRS and CAD (model 3). Model 3 presents a significant improvement in predictive ability compared with model 2 (*p* = 0.027 according to integrated discrimination improvement and *p* = 0.033 according to continuous net reclassification improvement).

We calculated the C index to compare the performance of MACE prediction among the different models. The C indices of the FRS-alone model, the FRS and CAD model, and the model adding DKK-1 to FRS and CAD were 0.64, 0.66, and 0.69, respectively (Figure 4). The model adding DKK-1 to FRS and CAD yielded a significant IDI of 0.012 (95% confidence interval (CI): 0.001–0.038, *p* = 0.027) and a significant NRI of 0.215 (95% CI: 0.009–0.370, *p* = 0.033) compared with the FRS and CAD model.

Table 2 presents the DKK-1 levels between the paired groups categorized by the association of risk factors. Higher DKK-1 levels were observed in patients with older age, a current smoking habit, lower insulin resistance reflected by the HOMA-IR index, lower HDL cholesterol, lower eGFR, higher triglycerides, higher platelet count, higher CRP, and higher NT-proBNP. All of the other assessed risk factors did not exhibit a significant association with DKK-1 levels.

We conducted multivariable Cox regression, and the results are presented in Table 3. The risk of MACEs was significantly higher in the high DKK-1 with CAD group than in the low DKK-1 without CAD group (hazard ratio = 10.640, 95% CI: 1.350–83.874, *p* = 0.025) after adjusting for age, sex, smoking status, hypertension, HOMA-IR index, total cholesterol, HDL cholesterol, triglycerides, eGFR, platelet count, CRP, NT-proBNP, UACR, and the use of statins.

## 4. Discussion

The main finding of the present study is that a combination of plasma DKK-1 levels and obstructive CAD acted as a better predictor of MACEs than plasma DKK-1 alone or obstructive CAD alone for a median of 4.8 years of follow-up in these patients. In accordance with our findings, the DKK-1 levels in serum have been reported to be a significant predictor of MACEs in patients after acute coronary syndrome. In a population-based cohort study, Klingenschmid et al. [19] reported that higher serum DKK-1 levels were significantly associated with a higher risk of composite cardiovascular events. However, these endpoint events not only included myocardial infarction, stroke, and transient ischemic attack, but also included angina pectoris, peripheral vascular disease, and revascularization procedures during a median follow-up duration of 15.6 years. Notably, a baseline history of prior cardiovascular disease was reported to be a significant factor of interaction in this association [19]. A strength of the present study is that plasma DKK-1 levels have a synergistic effect with obstructive CAD on the prediction of long-term MACEs in patients who have experienced angina.

There have been discordant results regarding the association between plasma DKK-1 and CAD in previous studies: a higher plasma DKK-1 level was reported in patients with stroke than in healthy controls [20]; however, plasma DKK-1 levels were not significantly different between patients with acute coronary syndrome and healthy controls [12]. Plasma DKK-1 levels were inversely associated with coronary atherosclerotic calcified plaque [21]. It has been reported that circulating DKK-1 levels vary by time in patients after an acute coronary syndrome [11]. In the present study, we assessed the plasma DKK-1 levels in patients in a stable condition after discharge, but not the levels measured during acute coronary syndrome. Notably, we found that patients with a higher plasma DKK-1 level with significant coronary obstruction had a significantly higher hazard ratio of MACEs than those with a lower plasma DKK-1 level without significant coronary obstruction, as determined using Cox regression analysis in the present study. In line with our findings, Wang et al. [22] reported that patients with higher plasma DKK-1 levels had a higher odds ratio of MACEs among patients with acute coronary syndrome using binary logistic regression analysis. 

The Wnt signaling pathway plays an important role in cardiovascular development and regeneration [23]. The aberrant regulation of Wnt signaling is involved in diverse cardiovascular diseases [8,24]. The activation of Wnt signaling pathway has been reported to improve left ventricular ejection fraction after MI in rats, and this benefit might result from a reduction in fibrosis after myocardial injury [25]. On the other hand, an overexpression of DKK-1 is associated with cardiovascular risk, including inflammation, endothelial dysfunction, and atherosclerotic development [26]. 

The endothelium is an important source of DKK-1 secretion. According to in vitro studies using human umbilical vein endothelial cells (HUVECs), the induced overexpression of DKK-1 in the endothelium could subsequently trigger monocyte adhesion to the endothelium, apoptosis of the endothelium, and a rupture risk for vulnerable plaques [10,27]. Moreover, DKK-1 is also released from activated platelets via thrombin receptor stimulation, and platelet-derived DKK-1 could induce the inflammatory pathway in HUVECs in vitro [12]. Platelet-derived DKK-1 also activated the intercellular adhesion of cultured cells to neutrophils [28]. Aspirin could inhibit the release of DKK-1 from activated platelets by thrombin receptor stimulation in an in vitro study [12]. Plasma DKK-1 levels were lower in heathy controls than in patients with diabetes, with increased platelet activity reflected by high urinary 11-dehydro-thromboxane B2, and aspirin was found to reduce plasma DKK-1 levels in patients with diabetes [29]. However, the plasma DKK-1 levels were not significantly different between the subjects who were using antiplatelet agents or not in a population-based cohort [19]. In the present study, the DKK-1 levels in plasma were not significantly associated with antihypertensive drug use or antiplatelet use.

Although angina is a clinical symptom of IHD [30,31], significant obstruction might not be detected on coronary angiography in some patients with angina [32]. In the present study, we enrolled patients with angina and suspected IHD based on noninvasive tests, including rest or exercise electrocardiography, stress nuclear imaging, and echocardiography. Only 29 (32.2%) of 90 female and 258 (66.8%) of 380 male patients had a significant obstruction detected on coronary angiography. In line with the Guidelines on Chronic Coronary Syndromes put forth by the European Society of Cardiology in 2019, the pretest probability of obstructive CAD is less than 27% in women and less than 52% in men [33]. Jespersen et al. [34] reported that no obstructive coronary lesion was detected in 65% of women and in 32% of men who had undergone coronary angiography for typical angina; however, these patients without obstructive CAD had an 85% increased risk of MACEs and a 52% increased risk of all-cause mortality compared with those without anginal symptoms. According to our results, a higher plasma DKK-1 level with obstructive CAD predicted a higher risk of MACEs compared with a lower plasma DKK-1 without obstructive coronary lesion. 

There are several limitations in the present study. First, we did not directly assess the underlying mechanism involved in DKK-1 and MACEs in patients with angina. Second, we did not examine microvascular dysfunction using invasive stress tests in the enrolled patients who did not present with significant coronary obstruction [35]. Third, although the baseline plasma concentration of DKK-1 was not associated with CAD, it might have a synergistic effect with coronary obstruction on MACE occurrence. However, this was an observational study, and we did not investigate the effect of any specific intervention on lowering plasma DKK-1 levels. Therefore, we cannot speculate that MACEs will be prevented by decreasing DKK-1 levels. Finally, we excluded patients with known DM. Although high glucose levels were detected in some patients at baseline, the results cannot be applied to the population with diabetes. In the present study, a lower plasma DKK-1 level was observed in patients with a HOMA-IR index ≥ 2 than in those with a HOMA-R index < 2. In line with our results, Qiu et al. [36] reported that plasma DKK-1 was significantly lower in Han Chinese individuals with diabetic retinopathy than in healthy controls. In contrast, Lattanzio et al. [29] reported that plasma DKK-1 was significantly higher in patients with type 2 diabetes than in healthy controls. Ali et al. [37] reported no significant association of serum DKK-1 with fasting glucose or the HOMA-IR index in men of African ancestry aged ≥ 40 years. The long-term effects of plasma DKK-1 on the population with diabetes should be further investigated.

## 5. Conclusions

Plasma DKK-1 levels in patients with angina and significant coronary obstruction were not significantly different from those in patients with angina but no significant coronary obstruction. However, higher plasma DKK-1 levels and obstructive CAD had a synergistic effect on predictions of a higher risk of MACEs in patients with angina during a median follow-up of 4.8 years.

## Figures and Tables

**Table 1:** The baseline characteristics of enrolled patients categorized by CAD and median plasma DKK-1.

	Low DKK-1 without CAD (n = 94)	High DKK-1 without CAD (n = 93)	Low DKK-1 with CAD (n = 141)	High DKK-1 with CAD (n = 142)	*p*	
Age (years)	56.9 ± 11.3	60.4 ± 11.9	60.6 ± 10.9	63.4 ± 11.5	<0.001	^a^
Male, n (%)	64 (68.1%)	62 (66.7%)	129 (91.5%)	125 (88.0%)	<0.001	
Current smoker, n (%)	21 (22.3%)	35 (37.6%)	40 (28.4%)	53 (37.3%)	0.044	
BMI (kg/m^2^)	26.5 ± 3.9	25.6 ± 3.7	26.4 ± 3.7	26.1 ± 3.9	0.104	^k^
Hypertension, n (%)	82 (87.2%)	83 (89.2%)	134 (95.0%)	136 (95.8%)	0.033	
Systolic BP (mmHg)	126.5 ± 18.4	127.4 ± 17.5	127.8 ± 16.3	128.5 ± 19.3	0.868	^a^
Diastolic BP (mmHg)	74.8 ± 11.0	74.4 ± 10.0	75.1 ± 11.1	73.8 ± 9.8	0.760	^a^
Fasting glucose (mmol/L)	5.2 ± 0.7	5.3 ± 0.6	5.4 ± 0.6	5.3 ± 1.0	0.092	^k^
Fasting insulin (µIU/mL)	11.7 ± 10.2	11.5 ± 10.6	13.9 ± 17.3	11.9 ± 16.1	0.215	^k^
HOMA-IR index	2.7 ± 2.4	2.8 ± 2.8	3.4 ± 4.3	3.0 ± 5.8	0.166	
Total cholesterol (mmol/L)	4.6 ± 0.8	4.8 ± 0.9	4.1 ± 0.9	4.4 ± 1.1	<0.001	^k^
HDL cholesterol (mmol/L)	1.3 ± 0.3	1.3 ± 0.3	1.2 ± 0.3	1.2 ± 0.3	<0.001	^k^
Triglycerides (mmol/L)	1.5 ± 0.9	1.8 ± 1.2	1.4 ± 0.7	1.6 ± 0.9	0.042	^k^
Framingham Risk Score	12 ± 4	14 ± 5	14 ± 4	15 ± 4	<0.001	^k^
eGFR (mL/min/1.73 m^2^)	91.7 ± 29.1	82.8 ± 23.7	85.6 ± 24.6	76.2 ± 24.1	<0.001	^k^
Platelet count (10^9^/L)	207 ± 57	219 ± 56	199 ± 47	207 ± 53	0.095	^a^
C-reactive protein (mg/L)	1.8 ± 1.9	2.3 ± 2.5	2.2 ± 2.3	3.0 ± 2.7	0.003	^k^
NT-proBNP (pg/mL)	1907 ± 4654	1261 ± 3193	1214 ± 3208	1484 ± 3153	0.085	^k^
UACR (mg/g)	24.5 ± 84.1	40.8 ± 135.7	47.0 ± 148.7	44.6 ± 132.3	0.068	^k^
Statins, n (%)	10 (10.6%)	20 (21.5%)	81 (57.4%)	73 (51.4%)	<0.001	

Categorical data are expressed as numbers (with percentages) and were examined by chi-square test. Continuous data are reported as mean ± SD: ^a^ presents ANOVA and ^k^ presents Kruskal–Wallis test. Major adverse cardiac events (MACEs) present a composite of all-cause death, nonfatal myocardial infarction, and nonfatal stroke. Abbreviation: BMI = body mass index, BP = blood pressure, CAD = coronary artery disease, DKK-1 = dickkopf-1, eGFR = estimated glomerular filtration rate, HDL = high-density lipoprotein, HOMA-IR = Homeostasis Model Assessment of Insulin Resistance, NT-proBNP = N-terminal pro-brain natriuretic peptide, SD = standard deviation, and UACR = urine albumin to creatinine ratio.

**Table 2:** Plasma DKK-1 concentrations between the groups categorized by risk factors.

	Group	Patient Number	DKK-1 (pg/mL)	*p*
Age	<60 years	(n = 221)	588 ± 194	0.021
	≥60 years	(n = 249)	628 ± 183	
Sex	Female	(n = 90)	614 ± 184	0.775
	Male	(n = 380)	608 ± 190	
Smokers	No	(n = 321)	595 ± 190	0.015
	Yes	(n = 149)	640 ± 184	
Hypertension	No	(n = 35)	602 ± 201	0.830
	Yes	(n = 435)	610 ± 188	
BMI	<27 kg/m^2^	(n = 311)	620 ± 185	0.089
	≥27 kg/m^2^	(n = 159)	588 ± 194	
Systolic BP	<130 mmHg	(n = 260)	599 ± 189	0.210
	≥130 mmHg	(n = 210)	621 ± 188	
Diastolic BP	<85 mmHg	(n = 395)	614 ± 194	0.230
	≥85 mmHg	(n = 75)	585 ± 160	
Fasting glucose	<5.56 mmol/L	(n = 345)	611 ± 188	0.750
	≥5.56 mmol/L	(n = 125)	604 ± 190	
HOMA IR index	<2	(n = 209)	635 ± 174	0.007
	≥2	(n = 261)	588 ± 197	
Total cholesterol	<4.14 mmol/L	(n = 198)	608 ± 202	0.923
	≥4.14 mmol/L	(n = 272)	610 ± 179	
Low HDL cholesterol *	No	(n = 331)	594 ± 184	0.009
	Yes	(n = 139)	644 ± 197	
Triglycerides	<1.7 mmol/L	(n = 322)	597 ± 186	0.041
	≥1.7 mmol/L	(n = 148)	635 ± 193	
eGFR	≥60 mL/min/1.73 m^2^	(n = 380)	592 ± 183	0.001
	<60 mL/min/1.73 m^2^	(n = 90)	681 ± 196	
Platelet count	<203 (10^9^/L)	(n = 235)	582 ± 173	0.002
	≥203 (10^9^/L)	(n = 235)	636 ± 200	
C-reactive protein	<1.393 mg/L	(n = 235)	580 ± 177	<0.001
	≥1.393 mg/L	(n = 235)	638 ± 196	
NT-proBNP	<328.7 pg/mL	(n = 235)	588 ± 177	0.017
	≥328.7 pg/mL	(n = 235)	630 ± 198	
UACR	<300 mg/g	(n = 453)	609 ± 191	0.778
	≥300 mg/g	(n = 17)	622 ± 133	
Use of statins	No	(n = 286)	605 ± 189	0.543
	Yes	(n = 184)	616 ± 189	
Use of antihypertensive drugs	No	(n = 60)	627 ± 204	0.426
	Yes	(n = 410)	606 ± 187	
Use of antiplatelet drugs	No	(n = 23)	597 ± 225	0.749
	Yes	(n = 447)	610 ± 187	

Data are reported as the mean ± standard deviation and were examined using the *t*-test. * Low HDL cholesterol means <40 mg/dL (1.0 mmol/L) in men or <50 mg/dL (1.3 mmol/L) in women. Abbreviations: DKK-1 = dickkopf-1, BMI = body mass index, BP = blood pressure, eGFR = estimated glomerular filtration rate, HDL = high-density lipoprotein, HOMA IR = homeostasis model assessment of insulin resistance, NT-proBNP = N-terminal pro-brain natriuretic peptide, and UACR = urine albumin to creatinine ratio.

**Table 3:** Cox proportional hazard regression models for the association between risk factors and major adverse cardiac events.

	Crude			Multivariable					
	HR	95% CI	*p*	HR	95% CI	*p*	HR	95% CI	*p*
Low DKK-1 without CAD	1.000			1.000			1.000		
High DKK-1 without CAD	8.185	(1.036, 64.690)	0.046	6.803	(0.857, 53.971)	0.070	7.370	(0.899, 60.408)	0.063
Low DKK-1 with CAD	8.622	(1.121, 66.313)	0.038	7.044	(0.913, 54.358)	0.061	6.291	(0.760, 52.056)	0.088
High DKK-1 with CAD	16.644	(2.257, 122.719)	0.006	12.100	(1.623, 90.182)	0.015	10.640	(1.350, 83.874)	0.025
Framingham Risk Score				1.098	(1.030, 1.170)	0.004			
Age (≥60 years)							3.258	(1.525, 6.960)	0.002
Male							1.268	(0.497, 3.235)	0.619
Current smoker							1.052	(0.544, 2.033)	0.881
Hypertension							1.191	(0.277, 5.125)	0.815
HOMA IR index ≥2							1.164	(0.616,2.199)	0.640
Total cholesterol ≥4.14 mmol/L							0.805	(0.425, 1.528)	0.508
Low HDL cholesterol *							2.095	(1.110, 3.955)	0.022
Triglycerides ≥1.7 mmol/L							0.728	(0.368, 1.439)	0.361
eGFR < 60 mL/min/1.73 m^2^							1.389	(0.719, 2.687)	0.328
Platelet ≥ 203 (10^9^/L)							1.149	(0.617, 2.141)	0.662
C-reactive protein ≥ 1.393 mg/L							1.773	(0.932, 3.374)	0.081
NT-proBNP ≥ 328.7 pg/mL							1.871	(0.560, 6.254)	0.309
UACR ≥ 300 mg/g							1.547	(0.771, 3.104)	0.219
Use of statins							1.181	(0.634, 2.201)	0.601

* Low HDL cholesterol means <40 mg/dL (1.0 mmol/L) in men or <50 mg/dL (1.3 mmol/L) in women. Abbreviations: CAD = coronary artery disease, DKK-1 = dickkopf-1, BMI = body mass index, BP = blood pressure, eGFR = estimated glomerular filtration rate, HDL = high-density lipoprotein, HOMA IR = homeostasis model assessment of insulin resistance, NT-proBNP = N-terminal pro-brain natriuretic peptide, and UACR = urine albumin to creatinine ratio.

## Data Availability

The data presented in this study are available on request from the corresponding author.
